# The Life History of Aneurisms

**Published:** 1895-08-03

**Authors:** A. Pearce Gould

**Affiliations:** Senior Assistant Surgeon to the Middlesex Hospital


### The Life History of Aneurisms.

By A. Pearce Gould, F.R.C.S., Senior Assistant Surgeon to the Middlesex Hospital.

The gravity of aneurismal disease arises mainly from two facts: (1) the important relations of the main arteries, and (2) the great weakening of the arterial wall usually attending the aneurismal dilatation. Any tumour forming in the position of any one of several of the arteries of the body must be serious from the pressure it exerts upon neighbouring structures. This element of danger pertains to aneurisms whatever their nature, and it is the only one associated with the so-called fusiform or tubular variety, in which the coats of the artery are not destroyed, but still form an impenetrable barrier to the escape of the blood. This is the great fact which not only justifies but compels the separation of these from the sacculated aneurisms; it is not a question of form or outline, but of nature, and therefore of danger. When fusiform aneurism occurs in an artery remote from structures of primary vital importance, it is not a very serious disease, and is usually untreated. Sacculated aneurism, on the other hand, is always characterised by a weakening of the arterial wall and a grave danger of rupture of the sac and escape of the blood.

Nature has two ways of guarding against the rupture of an aneurism; the first is the formation of clots within the sac, and the other is the strengthening of the sac on its outer side. Each of these processes is worthy of special study.

The formation of clots within an aneurism is due to the contact of the blood in the aneurism with the inner surface of the sac, or of its lining of previously-formed coagulum. This causes the disintegration of the cells containing the fibrin-ferment, and then the formation or separation of fibrin. It is not known what precise condition of sac leads to this separation of fibrin, and in some of the worst cases, where the interior of the sac appears to be very different from the smooth inner surface of the tunica intima of the normal artery, there may be found very little or no clot. The clot is of the highest importance. In the first place, by the substitution of solid clot for fluid blood, the total pressure on the sac is reduced, and hence the tendency to enlargement of the aneurism, and to stretching, thinning and rupture of its wall, is diminished. In the second place, it is through these clots that the natural cure of the aneurism is sometimes effected. If the clot is not only pressed against the sac, but kept at rest there, plasma cells pass into it from the sac and organise it into fibrous tissue which is inseparably blended with the sac, and which it strengthens on its inner aspect. By the repetition and continuance of this process the entire sac may be occluded and a natural cure be effected. It is specially important to observe that this organisation of the clot only takes place when the clot is left at rest in the sac. If the blood continues to be propelled with great force into the aneurism the clot is moved more or less over the sac, and its organisation then becomes impossible. The size and position of the mouth of the aneurism, and the configuration of the sac, will greatly modify the force and direction of the blood current in the sac, and upon this will depend the extent and the position of clots in the sac. It sometimes happens that the fibrin is deposited in concentric layers, quite uniformly, but at other times it is found in localised positions in the sac, and this may lead to great alterations in its size and antler.

But not only may the sac of an aneurism be strengthened by new tissue deposited on its inner surface, it may also be supported on the outer surface by pre-existing or new tissue. When the sac of an aneurism compresses a bone we usually find the bone speedily absorbed, but until that purpose is fully accomplished the bone is a substantial addition to the strength of the sac. In other cases a fascia is stretched so firmly over the aneurism that as a result the tense fascia forms practically a second sac outside the first and protects the patient against the rupture. The more common process, however, is for the tissues in the immediate neighbourhood of the aneurism to be first of all displaced by it in its growth, and then united to its outer surface by the chronic inflammatory products that are poured out around it. This, then, is the second great conservative process which is employed.

On the one hand, then, we have the pressure of the blood within the sac of the aneurism, tending to rupture the latter, and on the other hand we have two processes at work by which the sac may be thickened on its innermost surface, and at the same time thickened on its exterior. If these two processes exactly balance each other the result is a stationary aneurism or one that makes quite slow progress. If the conservative tendency predominates spontaneous cure results; if the aggressive tendency is the stronger, the aneurism enlarges. With the progressive enlargement of the sac its rupture becomes imminent. This rupture may be external or internal, and, if the latter, it may be into a mucous canal such as the œsophagus or bronchus, or into a serous sac such as the pericardium, pleura, peritoneum, or arachnoid, or into the planes of celluloid tissue in a limb or behind a serous cavity. In the case of rupture through the skin or into a serous cavity a sudden burst occurs, and a profuse hæmorrhage quickly closes the scene. But in the case of rupture into a mucous cavity the burst takes place gradually, and at first is a mere pinhole, which soon fills with clot, then enlarges, and allows of the escape of more blood, and so on until at last a fatal bleeding occurs. In the rupture into cellular tissue the same difference is seen; in some cases a large rent in the sac occurs and a widespread extravasation of blood results, but in others the yielding of the sac is at first at a minute orifice through which the blood only slowly leaks. Lastly, it is very important to remember that at some spot in the wall of a fusiform aneurism a thinning of the tissues may occur and introduce all the dangers inherent to a sacculated aneurism, and that in a sacculated aneurism the process of natural cure may occur at one time or at one place, and that of extension and rupture at another time or place.

